# Clinical Term Normalization Using Learned Edit Patterns and Subconcept Matching: System Development and Evaluation

**DOI:** 10.2196/23104

**Published:** 2021-01-14

**Authors:** Rohit J Kate

**Affiliations:** 1 Department of Computer Science University of Wisconsin-Milwaukee Milwaukee, WI United States

**Keywords:** clinical term normalization, edit distance, machine learning, natural language processing

## Abstract

**Background:**

Clinical terms mentioned in clinical text are often not in their standardized forms as listed in clinical terminologies because of linguistic and stylistic variations. However, many automated downstream applications require clinical terms mapped to their corresponding concepts in clinical terminologies, thus necessitating the task of clinical term normalization.

**Objective:**

In this paper, a system for clinical term normalization is presented that utilizes edit patterns to convert clinical terms into their normalized forms.

**Methods:**

The edit patterns are automatically learned from the Unified Medical Language System (UMLS) Metathesaurus as well as from the given training data. The edit patterns are generalized sequences of edits that are derived from edit distance computations. The edit patterns are both character based as well as word based and are learned separately for different semantic types. In addition to these edit patterns, the system also normalizes clinical terms through the subconcepts mentioned within them.

**Results:**

The system was evaluated as part of the 2019 n2c2 Track 3 shared task of clinical term normalization. It obtained 80.79% accuracy on the standard test data. This paper includes ablation studies to evaluate the contributions of different components of the system. A challenging part of the task was disambiguation when a clinical term could be normalized to multiple concepts.

**Conclusions:**

The learned edit patterns led the system to perform well on the normalization task. Given that the system is based on patterns, it is human interpretable and is also capable of giving insights about common variations of clinical terms mentioned in clinical text that are different from their standardized forms.

## Introduction

Clinical terms mentioned in clinical notes are not always in their standard forms as listed in standardized terminologies or ontologies. The use of synonymous words, abbreviations, syntactic variations, morphological alternations, and spelling variations are some common reasons clinical terms may be mentioned differently in clinical notes [1]. For example, a clinical note may mention “diffuse inflammatory reaction”, but a standard terminology resource such as Unified Medical Language System (UMLS) [2] may list the same clinical concept as “diffuse inflammation” or “inflammation diffuse”. As another example, a clinical note may mention “allergy to ferrous sulphate”, but the terminology may mention “allergy to ferrous sulfate”. Although a resource such as UMLS includes many synonyms for clinical terms, it does not exhaustively cover them. For example, neither of the 2 example mentions is listed in UMLS. In addition to the type of variations indicated earlier, mentions of clinical terms in clinical notes may have variations because of the writing conventions, or style of the medical center or simply because of typographical errors.

It is important, however, to map clinical terms mentioned in clinical notes to their corresponding concepts in a standard terminology for automated downstream applications, such as coding, biosurveillance, or clinical decision support, as well as for enabling portability of information across different medical centers. This task of mapping a clinical term mention to a standard terminology is called clinical term normalization. It is not trivial to automate this task because of all the possible variations of clinical terms mentioned earlier. For example, we found that in the test data set of the Medical Concept Normalization (MCN) corpus [3], only 62.37% of clinical terms matched exactly to the clinical terms listed in UMLS. There have been several approaches developed to automatically normalize clinical terms. Some of them use string-matching rules or approximations [4,5]. Other approaches cast clinical term normalization as an information retrieval [6] task and match clinical terms based on measures such as cosine similarity between their words [7,8]. More recently, machine learning methods have been employed for the clinical term normalization task [9,10], including deep learning–based methods [11-13]. Machine learning–based approaches for normalization have been shown to be more robust and accurate.

Previously, most clinical term normalization systems were evaluated on the benchmark data set of SemEval 2014 Task 7 [14], which had been previously used for the shared task of ShARe/CLEF eHealth Evaluation Lab 2013 [15]. However, this data set was designed for the combined task of information extraction [16] and clinical term normalization. In addition, it was restricted to clinical terms of only “disease and disorder” semantic type. Recently, a new corpus, called MCN [3], was created exclusively for the clinical term normalization task, which also includes clinical terms of other semantic types. This corpus was provided as the data set for 2019 n2c2 Track 3 [17], a shared task for clinical term normalization. In this paper, we describe our system that we had submitted for this shared task. This system is based on our earlier work [9,18] in which edit patterns to normalize clinical terms were automatically learned from the synonyms from UMLS. In this study, we extended that approach to also learn word-based edit patterns in addition to character-based edit patterns. We also extended it to learn patterns from the training data besides learning from UMLS. Previously, the approach had been evaluated only for the “disease and disorder” semantic type using the SemEval 2014 Task 7 data set. In this study, we evaluated it on other semantic types using the MCN data set. Besides the learned edit pattern–based component, our system includes a new subconcept matching–based component for normalization. Our system also includes a disambiguation component to choose the best concept for normalization in case there are multiple potential concepts.

Our system, UWM, achieved an accuracy of 80.79% on the test data set of the MCN corpus, which ranked sixth among the 33 system submissions and was behind by only 1.15% (absolute) to the second ranked system (81.94%) and was well above the mean (74.26%) and the median (77.33%) of all the participating systems [17]. The top system scored 85.26% and used a massive end-to-end deep learning architecture. An advantage of our method, however, is that because it is pattern based, it is easy to interpret how the system does normalization and it also provides insights into common variational patterns found in clinical terms. It also does not require heavy computational resources that are typically required for deep learning–based methods.

The objectives of this study are (1) to develop a clinical term normalization system using edit patterns learned automatically from synonyms of clinical terms and improve it further through subconcept matching and (2) to evaluate the system and its components on the MCN data set of clinical term normalization.

## Methods

This section describes our system for clinical term normalization and the data set used for its evaluation.

### Data Set

We used the MCN corpus [3], which was provided to the participants of the 2019 n2c2 Track 3 shared task. This data set consists of 100 discharge summaries, which is a subset of the clinical notes that were originally used for the fourth i2b2/VA shared task [19] and has now become a benchmark data set for clinical named entity recognition. These clinical notes were obtained from the Partners HealthCare and Beth Israel Deaconess Medical Center. In the MCN corpus of 100 discharge summaries, the spans of the concept mentions were manually annotated with their concept unique identifiers (CUIs) from UMLS (2017 AB version). The CUIs were restricted only to the 2 vocabularies of SNOMED CT (US version) and RxNorm (for medications), as present in the UMLS Metathesaurus. The concepts were medical problems, treatments, and tests. The data set was divided into training and test sets, each with 50 discharge summaries; the training data set had 6684 mentions, and the test data set had 6925 mentions. There were a total of 3792 unique CUIs. For the normalization task, the character spans of the mentions in the discharge summaries were provided, and the systems were required to identify their CUIs.

A few guidelines that were used for the annotation process of this data set are worth mentioning. If a mention span could not be mapped to any CUI, the annotators assigned multiple CUIs to that mention whenever possible. For example, “left breast biopsy” could not be normalized to any existing concept in SNOMED CT; hence, the annotators instead annotated “left” and “breast biopsy” to their respective 2 CUIs by identifying the largest span that could be normalized [3]. For the normalization task, the character spans of “left” and “breast biopsy” were separately provided to be normalized independently. Ties were resolved during the adjudication stage for consistency; for example, alternatively, one could have annotated “left breast” and “biopsy”. Theoretically as well as ideally, one could convert such compositional mentions into their postcoordinated concepts in SNOMED CT [20,21], but this was not done for this data set. The mentions for which the above compositional concept annotation strategy did not help were annotated as CUI-less. There were 2.70% (368/13,609) CUI-less mentions in the entire corpus.

A mention could be over multiple spans, which was indicated in the data set through multiple character spans but was assigned a single CUI. For example, for the mention “left atrium is moderately dilated”, there will be two separate character spans—one for “left atrium” and one for “dilated”—and hence the clinical term to be normalized will be “left atrium dilated” that will be assigned a single CUI, given that the concept exists in SNOMED CT.

We want to point out that although the MCN corpus as well as the 2019 n2c2 task has been called “concept normalization”, the task is, in fact, “term normalization” because the terms are being normalized and not the concepts. In the context of SNOMED CT, concept normalization means normalizing a concept to its standard form in SNOMED CT [22]. In SNOMED CT, a concept is represented in terms of its relations with other concepts, and there is often more than one way to represent a concept. Thus, concept normalization is a task in which a SNOMED CT concept is represented in terms of its relations in a standardized, unique way [23]. Concept normalization is, in fact, independent of any clinical term used to express the concept. On the other hand, term normalization means normalizing a clinical term to its standardized form in a terminology. Hence, in this paper, we will call the task “clinical term normalization” instead of “clinical concept normalization”.

### Clinical Term Normalization System

**Figure 1:**
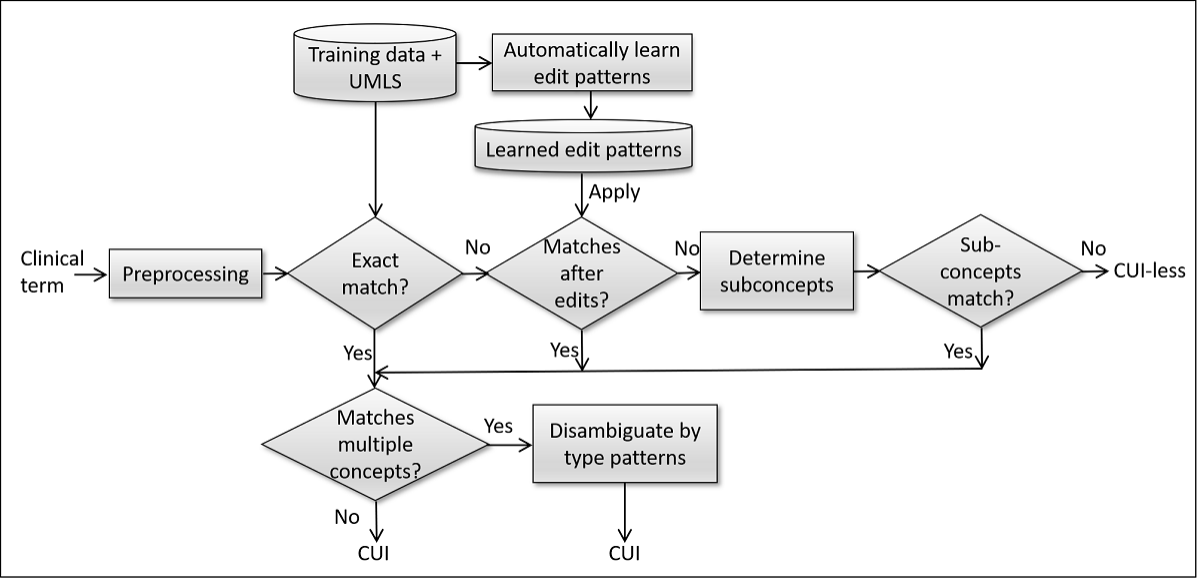
Overview of the clinical term normalization system. CUI: concept unique identifier; UMLS: Unified Medical Language System.

Given a mention of a clinical term in a clinical text, the task of clinical term normalization is to map it to its corresponding concept in the terminologies of SNOMED CT or RxNorm by assigning it the UMLS CUI or assigning it *CUI-less* if there is no such corresponding concept in the terminologies. This section describes our system for clinical term normalization. Figure 1 gives an overview of this system.

### Preprocessing

An input clinical term is first lowercased because our entire system works only with lowercased characters. Next, some common words are removed which were known to have been included in the mention spans because of the i2b2 complete noun/adjective phrase annotation policy [19]. These common words included “a”, “an”, “the”, “his”, “her”, “patient”, “patient’s”, any”, “your”, “this”, “that”, and “these”. In addition, characters “’s”, “’d”, “-”, “’”, “>”, and “<” were also removed from the mentions.

### Exact Matching

Most mentions of clinical terms found in clinical text often exactly match the clinical terms already listed in UMLS. In addition, many clinical terms in the test data of the MCN corpus are common enough that they have already been mentioned and annotated in its training data. Hence, as a first step, our system tries to exactly match the input clinical term with the already annotated terms in the MCN training data as well as in UMLS. To match in UMLS, all the English language synonyms of the concepts that are present in SNOMED CT and RxNorm are checked for equality match. In the implementation, this is done efficiently using a hash table. In the *Results* section, we report the accuracy of exact matching in only the training data, in only UMLS, and together in both of these.

Although exact matching seems straightforward and one would expect it to always lead to the correct answer, sometimes the same clinical term exactly matches with more than one concept. For example, “atrial fibrillation” is listed as a term for a “disease and syndrome” concept with CUI C0004238, and it is also listed as a term for “laboratory result or test” concept with CUI C0344434. The latter is in the sense of a finding of electrocardiogram. Hence, the exact matching process would match both the concepts, thus leading to 2 possible CUIs as output. This type of ambiguity of multiple possible output CUIs commonly occurred in this data set, not just in the exact matching step but also in the subsequent steps of the system. Hence, we included a disambiguation component in our system that is described later.

### Automatically Learned Edit Patterns

If the input clinical term does not exactly match either in the training data or in UMLS, then our system tries to normalize it by editing it based on the common patterns of variations of clinical terms that are learned automatically from known synonyms of clinical terms. This method for normalization was introduced in our previous work [9], in which it was tested only for the clinical terms of “disease and disorder” semantic type for the SemEval 2014 Task 7 data set [14]. For 2019 n2c2 Track 3, we adapted this method in 3 ways—first, in addition to “disease and disorder” semantic type, now it also learns patterns for all other remaining semantic types present in these data; second, in addition to character-based patterns, now it also learns word-based patterns; and third, in addition to UMLS, now it also learns patterns from the training data to learn variations that are specific to the given corpus. In the following section, we describe this method and the adaptations.

#### Edit Patterns

This method is based on the observation that often the clinical terms expressed in clinical notes have common variations from their mentions in standard terminologies; for example, they may not mention “nos” (not specified) at the end, they may mention “neoplasm” instead of “tumor”, or they may have an extra “s” for plural, or have a spelling variation such as “tumour” instead of “tumor”, etc. Often, exact matching fails because of such variations. The method is designed to automatically learn such common variations from the synonyms of clinical terms from a resource such as UMLS. Given a list of clinical terms and their synonyms, for every pair of synonyms, the method computes the Levenshtein edit distance [24] between them, which is the minimum number of edit operations of insertions, deletions, and substitutions that will convert one term into another. For example, converting “glycemic” to “glycemias” requires minimum of 2 edits—insert “a” after “i” and substitute “s” for “c”. It is not the edit distance but the sequence of edits that is important for our method. The sequence of edits can also be obtained through the Levenshtein edit distance computation. We call the sequence of edits along with the characters that remain unchanged as an *edit pattern*. For example, the edit pattern that changes “glycemic*”* to “glycemias” will be “BEGIN SAME g SAME l SAME y SAME e SAME m SAME i INSERT a SUBSTITUTE c|s END”. The pattern essentially says, “keep the characters same till ‘i’ then insert ‘a’ and substitute ‘s’ for ‘c’”. The “BEGIN” and “END” signify that the edit pattern is applied from the beginning of the term and ends at the end of the term. However, this edit pattern can only convert “glycemic” to “glycemias” that were already known to be synonyms and hence is not useful unless it is generalized to match other clinical terms. The method next generalizes the edit patterns.

#### Generalization of Edit Patterns

**Figure 2:**
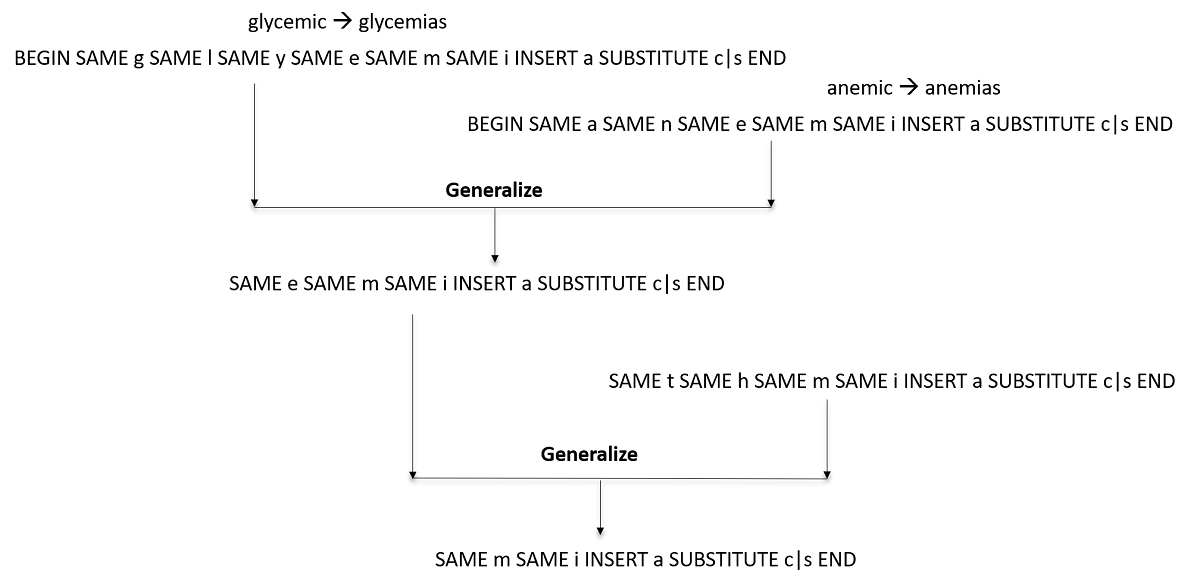
An illustrative example of how the method generalizes edit patterns by finding the longest contiguous common pattern that includes all the edit operations. In this example, it learns the edit pattern to convert clinical terms ending with “mic” to “mias”.

Given 2 edit patterns, their generalization is defined as the longest contiguous common pattern that includes all the edit operations. Thus, the generalization process generalizes over “SAME”, “BEGIN”, and “END” symbols. For example, given the edit pattern from the previous paragraph and the edit pattern “SAME a SAME n SAME e SAME m SAME i INSERT a SUBSTITUTE c|s END”, which converts *anemic* to *anemias*, the generalization will be the pattern “SAME e SAME m SAME i INSERT a SUBSTITUTE c|s END”, which says, “if ‘emic’ is at the end of a clinical term then convert it to ‘emias’”. This is shown in the top part of Figure 2. This generalized pattern can now apply to other clinical terms, for example, it can convert “ishemic” to “ishemias”. However, it will not convert “arrhythmic” to “arrhythmias” because the pattern expects an “e” before “mic”. The generalized patterns can be further generalized with other patterns using the same process of determining the longest contiguous common pattern. For example, once further generalized with “SAME t SAME h SAME m SAME i INSERT a SUBSTITUTE c|s END”, the new further generalized pattern will be “SAME m SAME i INSERT a SUBSTITUTE c|s END”, which will convert *arrhythmic* to *arrhythmias*. This is illustrated in Figure 2.

However, thus continuing to generalize will lead to overly general edit patterns, such as “SUBSTITUTE c|s” that says, “change every ‘c’ to ‘s’” that can change the meaning of a clinical term. Hence, there needs to be a way to gauge how good an edit pattern is and whether it is useful or overly general. In our method, this is done by counting the number of positives and negatives corresponding to every edit pattern. To compute these, the edit pattern is applied to the given list of clinical terms and their synonyms (eg, from UMLS). The number of times a clinical term is converted into one of its synonyms is counted as the number of positives. On the other hand, the number of times a clinical term is converted into another clinical term that is not its synonym (eg, a different concept in UMLS) is counted as the number of negatives. If the converted term is not a clinical term or it does not match in the list of clinical terms, then it is not included in the count of either positives or negatives. After computing the number of positives (p) and negatives (n), a score of p/(p+n+1) is assigned to the edit pattern, which is a simple form of m-estimate formula [25]. This score captures how accurate and how broadly applicable an edit pattern is in converting a clinical term into its synonym. Adding one in the denominator ensures that a pattern with a higher p will have a higher score even when n is zero. The patterns that are overly general will have a low score because they will have a high value of n. Good patterns will have a very high p value but a very low n value. Its score is used as the confidence of a learned edit pattern for normalizing a clinical term. We used a high threshold of 0.9 for the score, and only edit patterns with scores higher than 0.9 were included in the normalization system. We found through cross-validation within the training data that the method was not very sensitive to this threshold value, but it needed to be high for a good performance. An efficient algorithm to generate edit patterns using the method described above is given in a study by Kate [9].

We point out that the method to obtain edit patterns described earlier will always also generate a reverse pattern for each pattern. For example, if it generates a pattern to insert “s” in the end, then it will also generate a pattern to delete “s” in the end. This is because the synonyms are not considered in any order when generating the edit patterns; hence, each pair will be considered in both directions—generate the second from the first and generate the first from the second. As a result, the reverse of every edit pattern is also generated.

#### Applying Edit Patterns for Normalization

**Figure 3:**
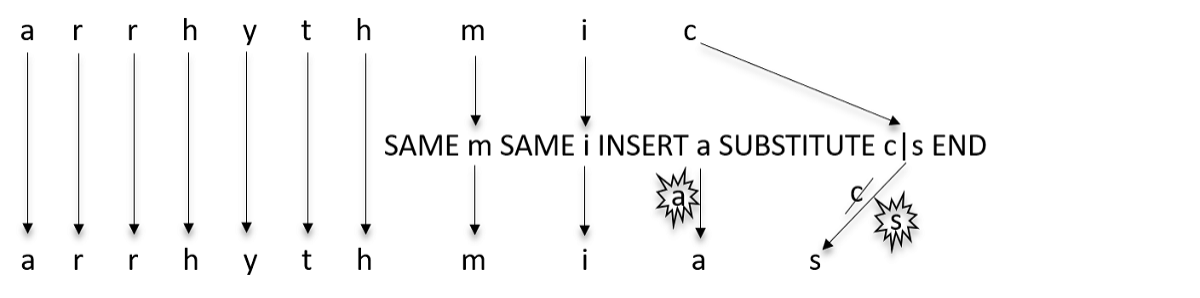
An illustration of how the edit pattern “SAME m SAME i INSERT a SUBSTITUTE c|s END” converts the clinical term “arrhythmic” to “arrhythmias”.

Given an input clinical term, an edit pattern is applied as follows. First, the system checks if the edit pattern matches the clinical term, that is, the clinical term is consistent with the presence of all the “SAME”, “SUBSTITUTE”, and “DELETE” characters as well as with the “BEGIN” and “END” symbols. For example, the edit pattern “SAME m SAME i INSERT a SUBSTITUTE c|s END” matches the clinical term “arrhythmic” because it has “mic” in the end. This is illustrated in Figure 3. If the edit pattern matches, then all its edit operations are applied at the matched location (in case an edit pattern matches at multiple locations within the clinical term, then each case is treated separately, although this rarely happens for a good edit pattern). In the previous example, “mic” will be changed to “mias”, hence converting the original clinical term “arrhythmic” to “arrhythmias”. Next, the system checks whether the resulting term is present in UMLS (or in its relevant portion, eg, within concepts of SNOMED CT and RxNorm) as one of the synonyms of the concepts. If so, the CUI of the corresponding concept is returned as the output of normalization. If the resulting clinical term does not match any synonym in UMLS, then the system moves on to match the next edit pattern. If multiple edit patterns match the clinical term, then all the corresponding CUIs are returned as the output; out of these, the best CUI is later selected by the disambiguation component. Given that our system only retains the edit patterns that have high scores, all the CUIs obtained by them are good potential candidates.

It should be noted that in this method, edit distance computation is used to generate edit patterns and not simply to find the closest term by edit distance because a close term by edit distance could often mean an entirely different concept. For example, the edit distance between “typical angina” and “atypical angina” is only one, yet the 2 clinical terms refer to 2 very different and, in fact, exactly opposite concepts. On the other hand, the edit distance between “cardiac sarcoidosis” and “heart sarcoid disease” is 12, yet they are synonyms. In our method, the edit pattern of “BEGIN INSERT a”, which inserts “a” in the beginning, will have many negatives and hence will receive a poor score. On the other hand, the edit pattern that changes “cardiac” to “heart” removes “osis” and adds “disease” will have many positives and very few or no negatives and hence will receive a high score. This shows that our method does not really depend on edit distance but only uses edit distance computation to generate edit patterns that are then generalized and judged for their goodness based on their numbers of positives and negatives.

#### Character-Based and Word-Based Edit Patterns

We described the method of learning edit patterns using examples in which characters were inserted, deleted, and substituted. However, sometimes, variations in clinical terms are simply due to the use of different words, such as “heart” instead of “cardiac”. Although these edits can also be expressed in terms of edits of characters, the generalization process over multiple patterns may lose such a pattern. Hence, in addition to character-based patterns, our method also directly learns word-based patterns such as “SUBSTITUTE cardiac|heart”. The method works in exactly the same way as described earlier, except that words instead of characters are treated as units of edits. In our method, words are tokens separated by whitespaces. In our results, we show the contribution of both types of patterns.

#### Edit Patterns From UMLS for Different Semantic Types

The clinical terms of different semantic types often exhibit different variations. For example, substituting “assay” for “measurement” is very common in clinical terms of “laboratory procedure” semantic type, whereas substituting “subcutaneous” for “intradermal” is very common in clinical terms of “clinical drug” semantic type. Hence, to capture such patterns efficiently, we applied our method of learning patterns separately to each of the 35 different semantic types of UMLS, which were the major semantic types of the clinical terms present in the MCN data set determined using its training set. For example, the top 5 semantic types in the training set were “disease or syndrome”, “pharmacologic substance”, “laboratory procedure”, “finding”, and “therapeutic or preventive procedure”. For each of the 35 semantic types, the method considers the concepts of that semantic type in UMLS and their listed synonyms and generates edit patterns. The patterns are both character based and word based, which are separately generated. We found that a maximum of 5000 concepts for each semantic type were sufficient to generate good patterns. Using more concepts did not help because the common variational patterns are easily learned from within that many concepts, and adding more concepts would only lead to additional learning of rare patterns that would not apply in the test set. Table 1 shows a few illustrative examples of learned edit patterns for 4 different semantic types. As the semantic types of test clinical terms are not given in the data set, edit patterns of all the semantic types are applied during normalization.

**Table 1:** Illustrative examples of edit patterns automatically learned from UMLS for a few semantic types and automatically learned from the training data. The first 4 and the last 2 edit patterns are word-based, whereas the remaining 4 edit patterns are character-based. The number of positives and negatives of each pattern are also shown.

Learned edit pattern		Positives	Negatives	Comment
**Clinical drug**				
	SUBSTITUTE intradermal|subcutaneous	133	0	Change “intradermal” to “subcutaneous”
	DELETE oral SUBSTITUTE tablet|tab	26	0	Change “oral tablet” to “tab”
**Diagnostic procedure**				
	SUBSTITUTE fibreoptic|fiberoptic	41	0	Spelling variation
	DELETE magnetic DELETE resonance SUBSTITUTE imaging|mri SAME of SUBSTITUTE both|bilateral	23	0	Change “magnetic resonance imaging of both” to “mri of bilateral”
**Laboratory procedure**				
	SUBSTITUTE k|c SAME o SAME c SAME y SAME t SAME e	54	0	Change “kocyte” to “cocyte”
	BEGIN SAME h DELETE a SAME e SAME m SAME o	52	0	Change “haemo” to “hemo” at the beginning of the clinical term
**Neoplastic process**				
	INSERT u SAME r SAME _space_	1148	2	Example: “tumor of”→“tumour of”
	SAME a SAME r SAME c SAME i SAME n SAME o SAME m SAME a DELETE s END	26	0	Delete “s” if the clinical term ends with “arcinomas”
**Training data**				
	SUBSTITUTE obs|finding	5	0	Change “obs” to “finding”
	INSERT on SUBSTITUTE o/e|examination	13	0	Change “o/e” to “on examination”

#### Edit Patterns From Training Data

The edit patterns learned from UMLS, as just described, capture the common universal patterns of variations in clinical terms. However, there are often patterns of variations in clinical terms that are unique to the genre of clinical notes or to the particular medical center from where the clinical notes were obtained. To learn these variational patterns, our method is also applied to the supplied training data of the MCN data set. To do this, the mentions of the clinical terms in the training data are added as additional synonyms of the UMLS concepts they were normalized to. These concepts (total 2311 unique) along with additional 3000 random UMLS concepts to drive the generalization process were used to learn edit patterns by the process described previously. In this case, we did not distinguish between different semantic types because there were not sufficient examples of each semantic type in the training data for the learning process. In the results, we separately evaluate the contribution of the edit patterns obtained from the training data. The last 2 rows of Table 1 show 2 illustrative edit patterns learned from the training data.

Of all the edit patterns thus obtained, only those with a score above the 0.9 threshold were retained as mentioned earlier. These were a total of 63,726 character-based and 22,832 word-based patterns. For a given input clinical term, each of the patterns is then applied as described earlier. If more than one CUI is obtained through this process, then the disambiguation component of the system (described later) is used to select the best CUI to output.

### Subconcept Matching

In case neither exact matching nor learned edit patterns could normalize a clinical term, then our system tries to normalize it using the subconcepts present in it. First, the method determines all the subconcepts present in the clinical term. This is done by considering all the subterms of the clinical term, which are all the contiguous word subsequences in the clinical term (ie, all n-grams), including of length one (ie, individual words). For each subterm, the method then checks if it matches in UMLS. The matched concepts are deemed to be the subconcepts of the clinical term and are represented in terms of their CUIs. For example, for the clinical term “nasal o2”, the method will find 2 subconcepts corresponding to the subterms “nasal” (CUI: C1522019) and “o2” (CUI: C4541402). Next, the method looks if there is any concept in UMLS that has exactly these subconcepts present. The subconcepts of a concept in UMLS are determined by finding the union of the subconcepts in each of its listed clinical terms in the same way by considering all its subterms. The UMLS concept of “oxygen administration by nasal cannula” has exactly the same 2 subconcepts corresponding to the subterms “nasal” (CUI: C1522019) and “oxygen” (CUI: C4541402). Hence, the clinical term “nasal o2” will be normalized to the UMLS concept of “oxygen administration by nasal cannula”. Note that in this case, exact matching would not have worked, and it is unlikely that an edit pattern would have captured this variation because it is not very common. Additionally, note that the method overlooks other subterms such as “administration by” and “cannula”, which do not correspond to any concepts in UMLS. If the clinical term cannot be normalized even after this method is applied, then the system outputs CUI-less.

Please note that this method is not the same as simple subterm matching, otherwise “o2” will not match “oxygen”. Instead, this method performs subconcept matching, which automatically considers the synonyms through the CUIs. One complication in this approach is that there could be multiple subconcepts (ie, multiple CUIs) corresponding to a subterm. For example, “o2” in addition to matching the concept with CUI C0030054 (the element oxygen) also matches the concept with CUI C4541402 (a military officer position). Hence, in our method, at least one match between the 2 sets of CUIs is deemed as a match of subconcept. In the abovementioned example, “oxygen” matches the CUI C0030054 (although it does not match the CUI C4541402), and hence, there is a match of the subconcept.

### Disambiguation

Each of the 3 normalization components described previously—exact matching, learned edit patterns, and subconcept matching—can lead to normalization to multiple concepts in UMLS. However, the normalization task, as set up for the MCN data set, is expected to output only one concept. Hence, the normalization system needs to disambiguate the concept whenever a clinical term is normalized to multiple concepts. We built a disambiguation component in our system, which is based on patterns of semantic types of the concepts to be disambiguated. We observed that it was often the case that when a clinical term was normalized to multiple concepts of a few semantic types, then the correct concept was frequently of one particular semantic type among them. Hence, we developed a method to automatically learn such rules from the training data. For all the clinical terms in the training data for which the system normalizes to multiple CUIs, it considers all combinations of different semantic types of those sets of CUIs. It then determines the combinations out of these for which the correct CUI is always of a particular semantic type. For example, it learned that whenever the multiple CUIs have semantic types of “finding”, “health care activity”, and “organism function”, the semantic type of the correct CUI was always “health care activity”. A total of 56 such patterns were automatically learned and were used during testing to resolve ambiguities. In case the ambiguity could not be resolved (ie, none of the patterns matched), then the first matched concept (effectively random) was output by default.

## Results

We experimentally evaluated the contributions of various components of our system on the task of clinical term normalization. All the results were obtained on the test data of the MCN corpus as provided for the 2019 n2c2 Track 3. As in the shared task, the performance was measured in terms of accuracy, that is, percentage of clinical terms that were normalized correctly—either to the correct CUI or correctly to CUI-less. There were a total of 6925 clinical terms to be normalized in the test data, of which 217 (3.13%) were CUI-less. In the following, we first show all the results obtained while using the disambiguation component. We later show how the results are affected if this component is not used.

Table 2 shows the results for the first component of our system that does exact matching. It achieved an accuracy of 76%. This shows that a large number of clinical terms can be normalized simply by exact matching. The next 2 rows of Table 2 show the contributions of exactly matching clinical terms only in the training data and only in UMLS. A large drop in accuracy can be seen in both cases. This shows that both the resources greatly contribute toward the combined accuracy and that neither is sufficient on its own to achieve good accuracy. Among the 2 resources, UMLS was found to be more important. However, it is clear that there are sufficient variations in clinical terms that are specific to this corpus and not present in UMLS. This could also be partly because of the conventions adopted by the creators of the MCN corpus for marking mentions in the clinical notes.

**Table 2:** Performance evaluation on the clinical term normalization task using only exact matching.

System	Accuracy (%)
Exact matching (training data+UMLS^a^)	76.00
Exact matching (training data only)	57.91
Exact matching (UMLS only)	62.37

^a^UMLS: Unified Medical Language System.

In Table 3, we show the results of adding the normalization component to the system that uses learned edit patterns. The results when only character-based patterns and when only word-based patterns are used are shown in the next 2 rows. In the last 2 rows, the results are shown when the edit patterns are learned only from UMLS and when learned from the training data (the latter also includes some terms from UMLS as described before). All these results include exact matching results (with both UMLS and the training data).

**Table 3:** Results of the ablation study for the method using different types of learned edit patterns.

System (includes exact matching)	Accuracy (%)
All edit patterns	79.93
Character-based edit patterns	79.6
Word-based edit patterns	78.28
Edit patterns from UMLS^a^	79.88
Edit patterns from training data	78.56

^a^UMLS: Unified Medical Language System.

It can be observed from the table that learned edit patterns helped in increasing the accuracy from 76% to 79.93%. This also shows that the method of learned edit pattern generalizes beyond “disease and disorder” semantic type, for which it was originally developed and evaluated [9], and works for other semantic types. From the next 2 rows of the table, one can see that character-based patterns were more important than word-based patterns. However, on its own, each type of pattern also did well. This indicates that character-based patterns can often express what word-based patterns can express and vice versa. For example, deleting the word “nos” can also be expressed as deleting those 3 characters; and changing characters “mic” to “mias” can be directly expressed as changing the word “arrhythmic” to “arrhythmias” (although its number of positives and negatives will be different). However, character-based patterns can exhibit better generalization in some cases; for example, deleting “s” at the end to convert plurals to singulars can be learned easily in a character-based pattern, but word-based patterns will have to learn that separately for each word.

The last 2 rows of Table 3 show how the performance changed when patterns learned only from UMLS were used and when patterns learned from training data were used. The results indicate that patterns learned from training data add to the accuracy but only marginally (from 79.88 to 79.93). The 2 illustrative edit patterns shown in the last 2 rows of Table 1 were learned only from the training data and could not be learned from UMLS alone. However, patterns learned without a large part of UMLS led to a larger drop in accuracy (78.56%).

The results in Table 4 show the contribution of the subconcept matching component of the system. Each result includes the exact matching results. Subconcept matching by itself obtains 77.79% accuracy and in combination with edit patterns, it increases the accuracy from 79.93% to 80.79%. This shows that this component is helpful, although not as important as edit patterns. The accuracy of our full system was 80.79%, which was the official accuracy of our system in the 2019 n2c2 Track 3 as evaluated and reported by the organizers.

**Table 4:** Results showing the impact of the subconcept matching component of the system.

System (includes exact matching)	Accuracy (%)
Subconcept matching	77.79
Edit patterns	79.93
Edit patterns+subconcept matching	80.79

In Table 5, we show the performance gain obtained by leveraging the training data. The result shown in the first row was obtained when training data were not used either for exact matching or for learning edit patterns. The second row shows the results of the full system in which training data are used for both the purposes. It can be observed that using training data greatly helps. This indicates that the clinical terms mentioned in real-world clinical notes frequently differ from how they are listed in UMLS. This could be because of linguistic variations used in writing free text as well as because of conventions or the style of writing clinical notes specific to a genre or a medical center. The large drop in accuracy was mostly because of not doing exact matching in the training data as was already observed in Table 2. Not learning edit patterns from the training data reduced accuracy by only a small amount, as was previously seen in Table 3.

**Table 5:** Results obtained with and without using the training data.

System	Accuracy (%)
Without using training data	68.01
With using training data	80.79

**Figure 4:**
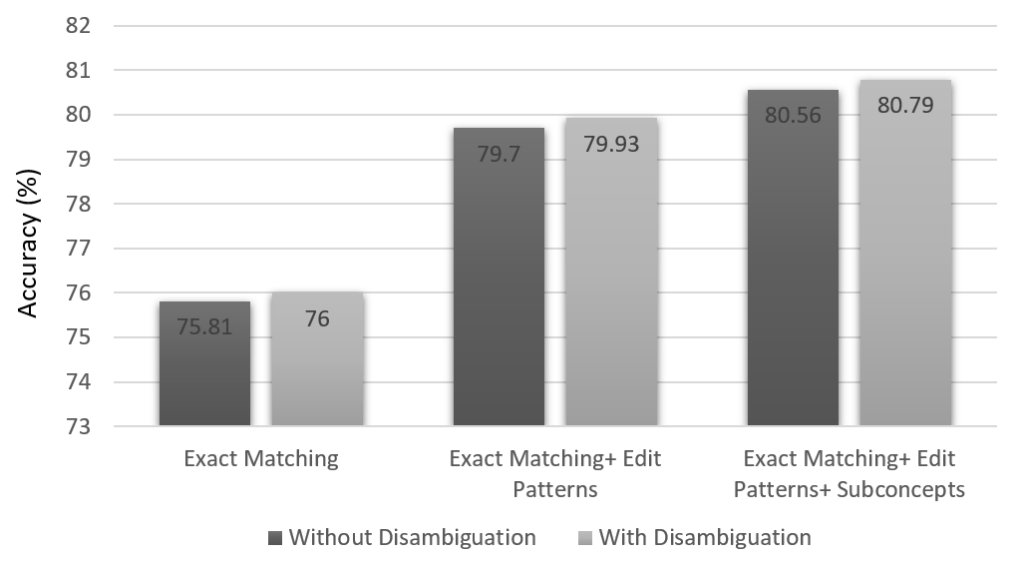
Accuracy (%) of the system on the Medical Concept Normalization data set evaluated by incrementally adding the normalization components with and without the disambiguation step.

All the results reported so far were obtained while using the disambiguation component of the system. The difference in performance because of this component is shown in Table 6 for different normalization components and their combinations. It can be observed that disambiguation consistently helps in each case but not by a large amount. The results obtained by incrementally adding the normalization components with and without the disambiguation step are graphically shown in Figure 4. To determine the upper limit for the disambiguation component, the results obtained using oracle disambiguation are shown in the last column of Table 6. In oracle disambiguation, the system’s normalization for a clinical term is considered correct if any one of the multiple CUIs it outputs is correct. One can see that the gap between accuracies of the system’s disambiguation and oracle disambiguation is very large (from 80.79% to 85.5%). This shows that when the system normalizes a term to multiple CUIs, then one of them is frequently correct, but it is not easy to determine which is the correct one. We also found that if semantic types of all input clinical terms are given, then the system achieves an accuracy of 83.64% without oracle disambiguation (in this case, the system ensures that the output CUI corresponds to the relevant semantic type). This shows that most of the ambiguity is between CUIs of different semantic types. For example, the name of a substance (eg, sodium) may correspond to the concept of the substance as well as to the concept of its measurement, and both will be of different semantic types. Similarly, many clinical terms could be normalized to a concept of "disease and syndrome" semantic type as well as to a concept of "laboratory or test result" semantic type that is used to determine that disease.

**Table 6:** Performance evaluation measured in terms of percent accuracy with and without the disambiguation component^a^.

System	Without disambiguation	With disambiguation	Oracle disambiguation
Exact matching	75.81	76.0	78.93
Edit patterns+exact matching	79.7	79.93	83.65
Subconcept matching+exact matching	77.62	77.79	83.31
Edit patterns+subconcept matching+exact matching	80.56	80.79	85.5

^a^The results of oracle disambiguation are also included in the last column for comparison.

## Discussion

### Principal Findings

We experimentally found that a majority of clinical terms can be normalized simply by exact matching in the training data and in UMLS. Both these resources contribute significantly when normalizing using exact matching. Beyond exact matching, we found that there are certain patterns common among synonymous clinical terms. These patterns are both character based and word based. We presented a method that learns such patterns automatically and uses them to edit clinical terms to match their known synonyms. Finally, we found that a few more clinical terms can be normalized by extracting their subconcepts and then matching these subconcepts.

The availability of training data was found to be critical in obtaining good accuracy thus indicating that variations of clinical terms found in clinical text could be specific to the type and source of clinical notes that may not have been captured in a general resource such as UMLS. We also found that many clinical terms in clinical text normalize to multiple clinical concepts. Although there are certain patterns based on semantic types that can help, in general, it is difficult to determine the correct concept when a clinical term normalizes to multiple concepts. This was a major source of error for our system. We note that the postadjudication interannotator agreement of the MCN data set was low (74.2%) [3], which also indicates that human annotators also faced the problem of multiple possible CUIs. It also shows that this data set is far from perfect, and automated systems will always have a certain amount of errors when evaluated on this corpus.

Besides ambiguity, we found a few more common sources of errors. Sometimes a clinical term mentioned in text would be in an implicit shortened form whose complete form would be inferable from its medical context to domain experts. For example, the text would mention “balloon” and mean (and thus normalize to) “balloon pump device”; similarly, it would mention “rhythm” and mean “finding of heart rhythm” or mention “alveolar” and mean “alveolar duct of lung”. However, our system would normalize only the shortened forms to their respective clinical concepts, thus leading to errors. Another source of error was the use of related words inside clinical terms that are not exactly synonyms; for example, the text would mention “upper lung field”, but it would normalize to “upper lobe of lung” or mention “airway protection” but normalize to “airway management”. Some errors were caused by subtle differences between concepts in SNOMED CT; for example, our system would normalize “left lower abdomen” to “entire left lower quadrant of abdomen”. but the correct answer was the concept “structure of left lower quadrant of abdomen”.

### Limitations and Future Work

As noticed earlier, the disambiguation component of our system has room for improvement. One limitation of our system is that it does not look at the surrounding context of the clinical term in the clinical note and treats the task of normalization independent of this context. Potentially, the context of a clinical term can help in determining its semantic type, which can then help in disambiguation. However, we also note that determining the semantic type of clinical terms is traditionally considered as part of the information extraction task and not the normalization task. For example, SemEval 2014 Task 7 required both information extraction and normalization in which the entities to be normalized were to be first extracted from clinical notes and were restricted to “disease and disorder” semantic type. Hence, the semantic type of the clinical terms to be normalized was already known, which reduced potential ambiguities. We also note that one could also modify the evaluation process to allow multiple CUIs for clinical terms when the corresponding concepts are equivalent or closely related. Another possibility is to provide rules for preferring one type of concepts over other types based on their semantic types or hierarchies in SNOMED CT or based on other criteria.

The learned edit patterns were found to be good at capturing sequential edits, but they could not capture if the edits were of a different kind. For example, to normalize “asthma–cardiac” to “cardiac asthma”, one needs to jumble the words, something that our edit patterns cannot capture (they will capture substituting each word with the other but that will not generalize to a pattern for jumbling the words that could match other clinical terms). In the future, patterns that capture such transformations could be learned from the data. Alternatively, a word-based similarity measure could also be used as is done in information retrieval [6]; however, it could also lead to incorrect normalization in other cases. Our method did not handle abbreviations of clinical terms separately. It either handled them through exact matching, if the abbreviations were mentioned as synonyms in UMLS or the training data, or through edit patterns that automatically learned abbreviations (eg, the edit pattern shown in the last row of Table 1). Given the prevalence of abbreviations in clinical text, in the future, using a dedicated component for abbreviation identification and disambiguation is likely to improve results [26].

Although our method may learn when a word can be substituted by another word, it does not consider word similarity which could potentially help in normalization. Incorporating word similarity in our method as captured through a suitable word embedding [27] will be an avenue for future work. The ontological structure of SNOMED CT in terms of its hierarchies and relations could also be leveraged for the normalization task in the future. For example, if the related concepts could be identified from the clinical term, then this can lead to finding the correct concept in SNOMED CT [21]. Edit patterns are used in our method to represent when 2 clinical terms can be normalized to the same concept. Another possibility for future work is to use a deep learning architecture to represent when 2 clinical terms could mean the same concept. For example, the neural network could take the edit pattern between 2 terms as input and learn to output whether the 2 clinical terms are synonymous or not. The network could be trained with the same examples from within the UMLS and training data as done in our approach.

### Conclusions

We presented a system for the clinical term normalization task. It uses edit patterns of both characters and words that are automatically learned from UMLS and the training data. The edit patterns capture how clinical terms can be edited to convert them into their synonyms to normalize them. These edit patterns are human interpretable and depict the common variations of clinical terms used in clinical notes. Our system also used the matching of subconcepts to normalize clinical terms. Our system achieved 80.79% accuracy on the MCN test data set. Whenever our system found multiple possible concepts to normalize a clinical term, often one of them was correct, but it was not easy to determine the correct concept as annotated in the data, which accounted for some loss in accuracy. Through ablation studies, we found that many clinical terms in the data set could be normalized by exact matching in UMLS and the training data, and normalization using learned edit patterns was the most important component for normalizing the rest of the clinical terms.

## Abbreviations

CUI: concept unique identifier
MCN: Medical Concept Normalization
UMLS: Unified Medical Language System
